# Multiplex PCR-Based Newborn Screening for Severe T and B-Cell Lymphopenia: The first Pilot Study in Turkey

**DOI:** 10.14744/SEMB.2020.09623

**Published:** 2021-01-12

**Authors:** Seyhan Kutlug, Medine Karadag Alpaslan, Gonca Hancioglu, Sariye Elif Ozyazici Ozkan, Didem Cemile Yesilirmak, Hasan Bulut, Canan Aygun, Gonul Ogur, Alisan Yildiran

**Affiliations:** 1. Department of Pediatric Allergy and Immunology, Faculty of Medicine, Ondokuz Mayis University, Samsun, Turkey; 2. Department of Medical Genetic, Ondokuz Mayis University Faculty of Medicine, Samsun, Turkey; 3. Department of Pediatrics, Division of Neonatology, Ondokuz Mayis University Faculty of Medicine, Samsun, Turkey; 4. Neonatology Service, Medikalpark Hospital, Samsun, Turkey; 5. Department of Statistics, Ondokuz Mayıs University, Samsun, Turkey; 6. Department of Pediatrics, Division of Pediatric Genetics, Ondokuz Mayis University Faculty of Medicine, Samsun, Turkey

**Keywords:** KREC, newborn screening, non-SCID T-cell lymphopenia, SCID, TREC

## Abstract

**Objectives:**

Severe combined immunodeficiency disease (SCID), non-SCID T-cell lymphopenia, and other primary immunodeficiency diseases with T-cell and B-cell lymphopenia have low the T-cell-receptor-excision circles (TRECs) and κ-deleting-recombination-excision circles (KRECs) levels that can be measured in dried blood spots (DBS) of the newborn. The incidence of SCID and non-SCID T-cell lymphopenia in Western societies has been reported by TREC screening of newborns as 1: 58,000 and 1: 7300, respectively. Since there is no similar study in our country, we aimed to perform the first pilot study of TREC and KREC screening of newborn for SCID and non-SCID T-cell lymphopenia in Turkey.

**Methods:**

The heel blood samples of newborns born between 1st October 2015 and 31st December 2016 at two major hospitals in our city were included in this study. TREC and KREC copies were determined by a multiplex quantitative PCR-based method from newborn DBS. Cutoff levels were used as 7 copies per DBS for TRECs and KRECs, 1000 copies for ACTB (internal control). Failed samples or abnormal results in measurements were tested the second time. An immunologist evaluated data of newborns with low TREC and KREC copies clinically and through the laboratory.

**Results:**

A total of 1960 DBS were tested. The results of 1856 newborns were evaluated. The low TRECs and/or KRECs levels were detected in 71 newborns (3.8 %). The low TRECs rate was 1.1 %. Preterm newborns have lower levels of TRECs and KRECs than term newborns (both p <0.0001). As a result of immunological research, we did not detect any SCID, but we detected 2 newborns with non-SCID T-cell lymphopenia (1:928). These 2 newborns were found to have frequent and severe infectious diseases or hypogammaglobulinemia in their clinical follow-up, although they did not have absolute lymphopenia.

**Conclusion:**

Non-SCID T-cell lymphopenia is common in our country than in western societies. TRECs and KRECs assay should be considered for routine NBS programs in our country. Studies involving more newborns should be conducted to detect SCID.

Primary immunodeficiency diseases (PIDs) are heterogeneous group of diseases characterized by congenital impairment of immunity, with high morbidity and mortality.^[1]^ Severe combined immunodeficiency disease (SCID) is one of the most severe forms of PIDs and have an immunological emergency.^[1, 2]^ In SCID, the main defect is T-cell scarcity or absence. B and NK-cells are affected at varying degrees. Affected infants lose their lives until the age of one if there is no rapid diagnosis and treatment in the first months of life.^[1-5]^ If these patients are detected early, curative treatment is possible.^[3, 5]^ Therefore, severe PIDs such as SCID are in compliance with the Wilson & Jungner principles which form the basis of newborn screening (NBS).^[2, 3, 6]^ The aim of NBS programs are to diagnose and successfully treat or cure diseases that are pre-symptomatic, but potentially serious or lethal in infancy, thereby reducing the number of preventable deaths and the medical costs.^[3, 6]^

A new screening biomarker for PIDs characterized by low or absent T or B-cells were investigated: T-cell receptor excision circles (TRECs) for T-cell lymphopenia and κ-deleting recombination excision circles (KRECs) for B-cell Lymphopenia.^[2, 7, 8]^ TRECs are small circular pieces of DNA which formed during T-cell receptor rearrangement in naïve T-cells.^[9]^ They are reduced or absent in SCID or other T-cell lymphopenia.^[2, 9]^ KRECs are small pieces of circular DNA, and are B-cell products produced during B-cell recovery. They were originally used for B-cell recovery following hematopoietic stem cell transplantation (HSCT).^[10]^ Later, they were used in NBS for B-cell lymphopenia.^[10]^ TRECs and KRECs are possible biomarkers for identifying disorders related to T and B-cell lymphopenia.^[2, 7, 8, 11]^ They can be extracted from DBS on Guthrie cards, and measured by real-time quantitative PCR.^[3, 7, 9]^ Primary immune-deficient patients with little or absent T and B-cells have very low or undetectable copy numbers of TREC or KRECs.^[2, 7, 8]^ It has been shown that the reduction of these DNA particles in the heel blood sample (HBS) of the newborn can be used to detect severe PIDs.^[2, 3, 9, 11]^

Pilot studies were completed the NBS for severe PIDs through TRECs and KRECs in some countries around the world, and its implementation has been successfully established in same countries.^[4, 12]^ NBS with TRECs is more implemented than KRECs screening.^[13]^ However, some serious PIDs can be detected from DBS through KRECs assay.^[10, 11]^ Therefore, TREC/KREC assay have many advantages over TREC screening alone.^[2, 7, 14]^ The prevalence of PIDs had been reported to be at 3 in 10.000 in a study from Turkey, although limited studies of incidence and prevalence for PIDs in Turkey.^[15]^ PID prevalence in our country is higher than western reports.^[12, 16]^ No pilot study has been published on the NBS for PIDs through TRECs and KRECs assay in our country so far. Therefore, we wanted to perform the first pilot study of NBS for severe PIDs or non-SCID T-cell lymphopenia by using TRECs and KRECs assay in a region of Turkey. In addition, we wanted to present information about clinical findings through the clinical follow-up of newborns with T-cell lymphopenia that we detected in NBS.

## Methods

### Study Design and Sample Collection

Heel blood sample of newborns born between 1^st^ October 2015 and 31^st^ December 2016 at two major hospitals in our city were included. Signed informed consent forms were obtained from the parents or guardians. Demographic and clinical data for all newborns were recorded. Four previously diagnosed PID patients and four healthy infants were used to test the reliability of the study. Mothers of infants who have health problems or receiving any immunosuppressive drugs were excluded from the study. This study was approved by local Ethical Committee (No: 2014/678) and the Turkish Ministry of Health, Public Hospitals Authority, Turkey (No: 54103609/604.02). The study was conducted in accordance with the ethical standards set forth in the Helsinki Declaration of 1964 and subsequent amendments or comparable ethical standards.

The heel blood samples were obtained at the time of other NBS tests, within the first 48-72 hours, on separate Guthrie cards. A DBS of 3.2 mm was taken from the Guthrie card for all newborns. Samples were stored in the fridge at +4 degrees after drying in the dark at room temperature.

### DNA Elution and Real-Time Quantitative Triplex PCR

A diagnostic neonatal screening kit for primary immunodeficiency diseases was used (ImmunoIVD, Stockholm, Sweden). The tests were performed with 96-well ready plates according to manufacturer instructions. After DNA elution, qPCR plate that contained specific primers for TREC, KREC, and ACTB (beta-actin) DNA sequences, as well as probes for target regions, was run for quantitative real-time PCR on the Applied Biosystems 7500 Fast Real-Time PCR System, USA.

### Quality Controls

ACTB was used as an internal control and reflected the successful extraction of DNA. TREC and KREC copy numbers were quantified by multiplex real-time PCR. Standards from plasmids, with a known number of copies, were used as internal controls, as well as T-cells, B-cells, and samples with known deficiencies in both, empty sample collection paper and no template control were used as external controls of the study. Calibration curves from plasmids were created by 10-fold sequential dilution. Besides, samples previously diagnosed with PID and healthy controls were used for confirmation of the assay. Since this method was being employed as the first time, the first 570 samples were studied twice for confirmation.

### Interpretation of the Copy Numbers

Before interpretation, all RT-qPCR assays were checked and confirmed for quality requirements of similar slopes and R^2^ values >0.97. Our negative and positive control samples were not enough to reach sufficient power analysis for defining cutoff values of TREC and KREC. Therefore, we couldn't calculate statistically significant cutoff value for our study, yet we used manufacturer instructions to set the cutoff value. Also, TREC cutoff values were generally reported at 7 and above in studies in different countries.^[12, 17]^ We used the cutoff values of TREC and KREC as 7 copies. In cases of ACTB copies ≥1000/μl and TRECs or KRECs copies <7 μl, the test was assumed positive or abnormal. In cases of TRECs and KRECs >7/μl, regardless of ACTB value, the test was assumed negative or normal. In cases of ACTB copies <1000/μl and TRECs or KRECs copies <7 μl, the test was assumed inconclusive. SCID is defined as absence of T-cells or CD3 T cells <300/μL and no or very low T-cell function (<10% of lower limit of normal) as measured by response to PHA. In non-SCID T-cell lymphopenia (clinical relevant T-cell lymphopenia), CD3 T-cells are 300-1500/μL.^[5, 18]^

### The Other Laboratory Measurements

After a positive screening result, lymphocyte subgroup analysis (Immunophenotyping) was performed for the newborns that could be reached. Immunophenotyping was performed with the following monoclonal antibodies: CD3, CD4, CD19APC, CD27 FITC, CD56 (BD Biosciences, Pharmingen, Germany). CD3 used for T-cells, CD19APC used for B-cells, CD4 used for T-helper cells, CD56 used for NK-cells. The B-cell subgroups (CD19, CD19CD27) were analyzed by using flow cytometry (BDFACS Calibur; BD Biosciences, San Jose, CA, USA). Memory B-cells defined as CD19CD27+ B-cells. Serum immunoglobulin G, A and M levels were measured using commercially available nephelometry kits (Dade Behring Marburg GmbH, Marburg, Germany).

Next-generation sequencing (NGS) for the PID was able to perform in only one newborn with a positive screening result. It has been performed with commercially available Inmunodeficiencias-GeneSGKit® (covers 200 genes responsible for PID) that is capable of sequencing samples at a minimum of 20x depth with 98% coverage and low variance on the Illumina MiSeq platform (Illumina, CA, USA).

### Statistical Analysis

Statistical analysis was performed using IBM SPSS v20 software (SPSS v20, Inc. Chicago, IL, USA). Kolmogorov-Smirnov test was used for the normality test, and it was seen that the normality assumption is not satisfied in all cases (p-value >0.05). Therefore, the Mann-Whitney U test was used for group comparison analyses, and a p-value below 0.05 was considered statistically significant.

## Results

A total of 1960 samples were tested. Failed samples in tests were studied second time by taken from the same Guthrie card. A total of 96 samples failed twice due to insufficient starting material on the additional screening card and were excluded from the study. Of the remaining 1856 newborns, 956 (52%) were males, 1638 (88%) were term, and 853 (46%) were born in OMU. Newborns with abnormal results in the first assay were tested again for the second time on the same Guthrie card. After the second abnormal result, the newborn was referred to a department of clinical immunology for immunological evaluation. Healthy controls are 1-year-old, 2 male and 2 female infants with no laboratories or clinical findings for PIDs. Four previously diagnosed PIDs patients were used as a positive control (Table 1).

**Table 1.** **Table 1.** Positive controls with known primary immunodeficiency diseases

**Age (y)/Sex**	**Clinical/ Genetic Diagnosis**	**The number of copies/DBS**		
	**ACTB**	**TRECs**	**KRECs**
14/M	Agammaglobulinemia	3.772	6	5
	No mutation in 200 gene			
30/M	TACI Deficiency	2.480	27	4
18/M	X-linked Agammaglobulinemia (Homozygous BTK)	6.201	21	1
<1/M	SCID, IL2RG mutation	4.470	1	248

M: male; TACI: The transmembrane activator and calcium-modulating cyclophilin ligand interactor; BTK: Bruton tyrosine kinase; SCID: Severe combined immunodeficiency; ACTB: Actin-beta; TREC: Tcell receptor excision circles; KRECs: κ-deleting recombination excision circles; DBS: dried blood spot; y: year; IL2RG: Interleukin 2 Receptor Subunit Gamma.

**Figure 1.**
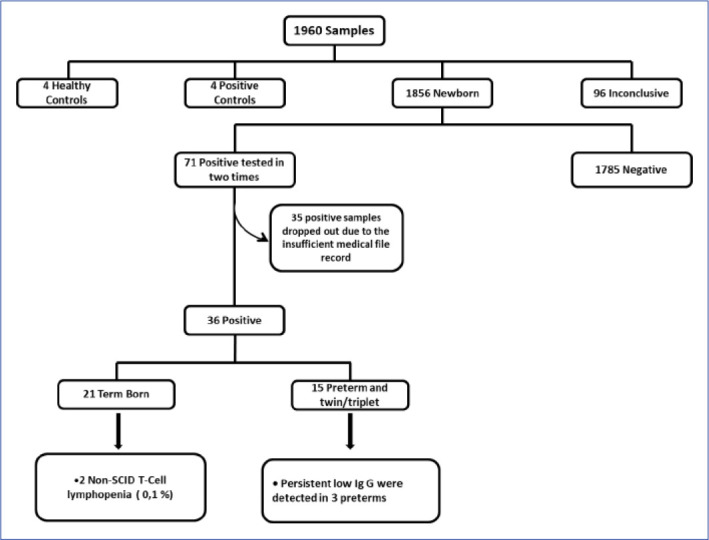
Summary of the study.

There were 71 (3.8%) positive samples according to TRECs or KRECs levels in the 1856 newborns **(Fig. 1)**. Among 71 positive samples, 10 newborns had low TRECs and KRECs, 12 newborns had low TRECs only, and 49 newborns had low KRECs only. Low TREC levels rate was 1.1 %. A second HBS could not be taken to detect the false positivity. Unfortunately, registries of 35 (49%) of the 71 positive samples could not be accessed in any way due to insufficient medical file records.

Laboratory and clinical data of the remaining positive 36 samples of term and preterm newborns are presented in the Table 2 and Table 3 respectively. We analyzed lymphocyte subgroups in 7 term newborns with positive screening results. In our cohort, we did not detect any SCID newborn. Among 1856 newborns, we found 2 (0.1%) term newborns with non-SCID T-cell lymphopenia. In addition, we found another term newborn with mild low T-cells. These 3 term newborns also had low NK-cells. Although these 3 newborns did not have absolute lymphopenia, they were found to have frequent and severe infectious diseases or hypogammaglobulinemia in their clinical follow-up. Of the infants without lymphocyte subgroup analysis, 4 infant had persistent hypogammaglobulinemia after 6 months of age, 3 of them were preterm (Table 3). Unfortunately, we could not reach the clinical follow-up information of these newborns.

**Table 2.** The clinical and laboratory characteristics of term newborns with positive screening results (n=21)

**Newborn ID/Sex**	**ACTB/TREC/KREC**	Lymphocyte and its subgroups, /mm^3^ (Normal range**)						**The other findings**
		**Age, in month/Lymphocyte**	**CD3+**	**CD4+**	**CD19+**	**CD19+ CD27+**	**CD56+**	
116/F*	2360/2/9	36/2900	1006	693	272	19	27	Late neonatal sepsis, Low IgG and IgM
			(1400-3700)	(700-2200)	(390-1400)	(45-175)	(130-720)	
163/M*	1188/3/6	36/2400	900	478	365	30	70	HFID, Normal immunoglobulins, Epilepsy,
			(1400-3700)	(700-2200)	(390-1400)	(45-175)	(130-720)	Cerebral palsy.
219/F	3224/6/17	28/3027	2209	1422	544	90	211	Normal immunoglobulins
			(1400-3700)	(700-2200)	(390-1400)	(45-175)	(130-720)
246/M*	1747/5/20	17/2899	1623	1188	1014	28	115	Late neonatal sepsis, IgG, IgA
			(2100-6200)	(1300-3400)	(720-2600)	(25-217)	(180-920)	
248/M	3167/24/6	35/4282	2997	1926	856	51	299	HFID, Epilepsy, Cerebral palsy, Normal
			(1400-3700)	(700-2200)	(390-1400)	(45-175)	(130-720)	immunoglobulins, Mutation in NFKB2 gene
480/M	1435/4/5	34/2748	1758	961	687	27	164	No HFID, Normal immunoglobulins, Epilepsy
			(1400-3700)	(700-2200)	(390-1400)	(45-175)	(130-720)	
590/M	2678/6/48	N/A	N/A	N/A	N/A	N/A	N/A	Maternal diabetes mellitus
686/M	1901/4/58	36/3220	N/A	N/A	N/A	N/A	N/A	No HFID, Low IgG, IgM and IgA
719/M	1895/16/3	N/A	N/A	N/A	N/A	N/A	N/A	N/A
749/M	3932/4/28	1/3720	N/A	N/A	N/A	N/A	N/A	N/A
874/F	1236/3/1	6/5200	3900	3068	1144	260	156	No HFID, Normal immunoglobulins
			(1900-5900)	(1400-4300)	(610-2600)	(25-217)	(160-950)	
1181/M	3475/5/19	N/A	N/A	N/A	N/A	N/A	N/A	Developmental retardation, lymphadenopathy
1184/M	2124/4/15	N/A	N/A	N/A	N/A	N/A	N/A	N/A
1202/F	3055/3/8	N/A	N/A	N/A	N/A	N/A	N/A	Dysmorphism, trigonocephaly
1208/F	1637/3/25	N/A	N/A	N/A	N/A	N/A	N/A	N/A
1228/M	1502/5/20	N/A	N/A	N/A	N/A	N/A	N/A	Normal immunoglobulins, midgut volvulus
1230/M	2467/4/24	N/A	N/A	N/A	N/A	N/A	N/A	Normal immunoglobulins, microcephaly, eczemaa
1234/M	2261/3/17	N/A	N/A	N/A	N/A	N/A	N/A	Transient neutropenia
1269/F	3064/5/5	N/A	N/A	N/A	N/A	N/A	N/A	Hearing loss
1299/M	1075/3/33	N/A	N/A	N/A	N/A	N/A	N/A	Neutropenia, eczema
1313/M	1664/6/22	1/2390	N/A	N/A	N/A	N/A	N/A	Anal atresia

* Newborns with low T-cells and B-cells according to analysis of flow cytometry and their lymphocytes subsets are in bold; ** Age-matched normal values for lymphocyte subgroups are taken from two references (Shearer WT et al. Lymphocyte subsets in healthy children from birth through 18 years of age: the Pediatric AIDS Clinical Trials Group P1009 study. J Allergy Clin Immunol. 2003;112(5):973-980. Duchamp M et al. B-cell subpopulations in children: National reference values. Immun Inflamm Dis. 2014;2(3):131-140); M: Male, F: Female, IgG: Immunoglobulin G, IgA: Immunoglobulin A, IgM: Immunoglobulin M, ACTB: Actin-beta, TRECs: T-cell receptor excision circles, KRECs: κ-deleting recombination excision circles, HFID: History of frequent infection disease, NFKB2: Nuclear factor kappa B2, N/A: Not available.

**Table 3.** The copy numbers and clinical findings of preterm newborns with positive screening results (n=15)

**Newborn ID/Sex**	**ACTB/TREC/KREC Copy numbers per DBS**	**Clinical Findings**
329/F	1963/5/6	Eosinophilia, Triplet
343/F	1338/54/4	Born weighing 600 g,
		Deceased
486/M	1338/15/5	Low IgG*, Septicemia,
		West syndrome
599/M	2187/6/8	Septicemia
718/F	1232/4/56	Neutropenia
745/F	1483/11/2	Low IgG *, Neutropenia,
		Twin
830/M	1224/1/3	Low IgG *, Midgut
		volvulus
970/F	1006/11/3	Died on the first day
1097/M	1324/5/8	Twin
1109/M	1548/6/15	Twin
1122/M	1516/46/3	Twin
1215/M	1060/6/1	Twin, Syndactily,
		Hellp syndrome
1223/M	1477/9/1	Twin
1331/M	1502/3/9	Twin
1581/M	1372/4/1	Twin

* Hypogammaglobulinemia persisted after 6 months; F: Female; M: Male; DBS: Dried Blood Spots; IgG: Immunoglobulin G.

ACTB, TRECs, and KRECs copy numbers per DBS were compared between term and preterm newborns (Table 4). Preterm newborns have lower levels of TRECs and KRECs than term newborns (both p<0.0001). Within the term newborn group, female newborns had significantly higher median KRECs levels than males (p=0.021) (Table 5). There was no significant difference between females and males for ACTB and TRECs levels. In preterm group, there was no significant difference between females and males in terms of ACTB, TRECs and KRECs (Table 5). Differences in DNA copies according to sex in all newborns with positive screening results were as in the term newborn group (Table 5).

**Table 4.** Comparison of ACTB, TRECs and KRECs copy numbers between term and preterm newborns.

**Markers**	**Term (n=1638)**	**Preterm (n=218)**	**p^a^**
ACTB	2945.075	2690.775	0.019^b^
		(112.54-29178.45)	(357.73-28868.74)
KRECs	26.995	20.875	0.000^c^
		(0.22-522.93)	(0.89-143.69)
TRECs	39.335	24.96	0.000^c^
		(0-956.85)	(0.62-345.83)

^a^Mann-Whitney Test; ^b^Significant difference at 95% confidence interval; ^c^Significant difference at 99.9% confidence interval.

**Table 5.** Comparison of ACTB, TREC, and KREC values in female and male newborns

**Markers**	**Term Newborns (n=1638)**			**Preterm Newborns (n=218)**			**All Newborns (n=1856)**		
	**Male (n=835)**	**Female (n=803)**	**p^a^**	**Male (n=121)**	**Female (n=97)**	**p^a^**	**Male (n=956)**	**Female (n=900)**	**p^a^**
ACTB	2953.65	2917.39	0.717	2408.70	2894.81	0.242	2911.43	2917.32	0.871
	(309.04-29178.45)	(112.54-14663.84)	(357.73-14479.71)	(404.66-28868.74)	(309.04-29178.45)	(112.54-28868.74)	
KRECs	25.61	28.62	0.021^b^	20.48	21.19	0.793	24.52	27.36	0.019^b^
	(0.22-315.27)	(1.37-522.93)		(0.89-143.69)	(2.38-119.59)		(0.22-315.27)	(1.37-522.93)	
TRECs	39.48	38.83	0.988	24.235	26.85	0.687	37.89	37.97	0.771
	(0-835.62)	(0.2-956.85)		(0.62-345.83)	(2.36-331.07)		(0-835.62)	(0.2-956.85)	

^a^Mann-Whitney Test; ^b^Significant difference at 95% confidence interval; *Minimum and maximum values were written in smaller punto in the parenthesis.

A term newborn with a positive screening result (#248 ID) had hypogammaglobulinemia at baseline. He had frequent respiratory infection diseases, as well as epilepsy and cerebral palsy. Lymphocyte subgroup and genetic analysis were performed simultaneously. His lymphocyte subsets were normal. Heterozygous c.2183T>G (p.Leu728Arg) missense mutation in nuclear factor-kappa B subgroup 2 (NFKB2) gene was detected in this infant. In his clinical follow-up, immunoglobulins were returned to normal. At the age of 3, there were no skin and hair abnormalities or signs of adrenal insufficiency.

## Discussion

In the present study, we used a combined TREC and KREC assay on Guthrie card to detect the incidence of the severe PIDs characterized by pronounced T-cell lymphopenia in a newborn cohort. Our study is the first one in our country in this aspect. We didn't found any SCID patient, yet we found the incidence of non-SCID T-cell lymphopenia as 0.1% (1:928) which is higher than the prevalence of PID (0.03%) reported in Turkey.^[15]^

It has been reported that the incidence of PIDs is increasing globally due to the NBS test for PIDs.^[13, 16, 19]^ While SCID incidence was previously reported as 1: 100.000, this ratio was determined as 1: 58.000 as a result of NBS through TRECs. ^[13]^ We may not be able to detect a SCID due to the small number of our newborn cohorts. As a result of NBS, the incidence of non-SCID T-cell lymphopenia in Western societies was reported between 1 : 15300 and 1 : 7300,^[13, 20, 21]^ but our results are indicating that this rate as higher in our country (1 : 928). In some countries, including our country, the calculated prevalence and incidence of PIDs is higher than in western societies.^[12]^

In 2005, TRECs assay was described in NBS for PIDs.^[9]^ This assay has been successfully applied in some societies and has been shown to be effective.^[20, 22]^ However, the TRECs screening method alone cannot detect all SCID types and other combined immunodeficiency diseases.^[2, 7, 14]^ KRECs assay has been developed for B-cell lymphopenia and monitoring B-cell recovery following HSCT,^[10]^ and has been used since 2011 to detect PIDs in newborns.^[11]^ The addition of KRECs assay to NBS can help to detect some PIDs that cannot be detected by TRECs assay alone, such as leaky SCID phenotype, delayed-onset adenosine deaminase deficiency, ataxia telangiectasia, Nijmegen breakage syndrome, inherited agammaglobulinemias, and purine nucleoside phosphorylase deficiency.^[8, 21, 23]^ In addition, the KREC assay can help distinguish some types of SCIDs from each other.^[7, 8]^ Some experts suggested that the inclusion of KRECs may increase the rate of false positivity and family anxiety, the number of re-testing and the costs.^[21]^ However, the inclusion of KRECs has been shown to not increase the rate of re-call and re-punch, and false positivity.^[13, 14]^ Therefore, it has been subsequently developed a triplex (multiplex) method that also includes KREC analysis to assess for potential B-cell lymphopenia from DBS.^[7]^ This combined method has also been shown to be cost-effective.^[16, 24]^ That's why we used the multiplex method, which measures TRECs and KRECs together, like recently published other studies.^[23, 25]^

Low TRECs and KRECs levels have been reported in preterms, and newborns with low birth weight.^[14, 21, 23, 26]^ Consistent with the literature, we found that the TRECs and KRECs levels in preterms were lower than in terms. However, there are also studies that did not find any difference in terms of KRECs levels between preterm and term.^[27, 28]^ The impact of gestational age and birth weight on TRECs was stronger compared to the KRECs.^[14, 26]^ This is in line with the B-cell maturation to precede T-cell maturation in human fetuses.^[29]^ Therefore, low KRECs are more important than low TRECs in preterms. Moreover, adding KRECs assay to the NBS for PID in preterms does not increase false positivity, it becomes more cost-effective to detect the newborns with positive screening.^[14]^ Preterm newborns have lower IgG levels than term newborns.^[30]^ Therefore, we think that additional KRECs measurement will be useful in the neonatal cohort including premature newborns. Hypogammaglobulinemia persisted after 6 months in our 3 out of 15 preterms with positive result. All of these 3 preterms had low KRECs, 1 had low TRECs. In the literature, there are studies reporting that TREC is not affected by sex as in our study,^[20]^ there are also studies reporting that TREC is higher in female newborns.^[20, 21, 31, 32]^ At the same time, we found the KREC copy number is higher in the female group, which we could not explain. There is no study in the literature on why the number of copies may change according to sex. Further studies should be done in this regard.

In the case of low or undetectable TRECs or KRECs values in the newborn, lymphocyte subgroup analysis should be performed.^[2, 5, 7]^ In our study, the rate of lymphocyte subgroup analysis is low. However, this rate was also low in other studies.^[13, 23]^ The most important reason for the low rate of performing both repeat and advanced analyzes in newborns with positive screening result is the lack of follow-up of the newborns.^[14, 25, 27]^ In our cohort, 3 newborns with low T-cells and NK-cells have frequent and severe infectious diseases or hypogammaglobulinemia in their clinical follow-up, although they did not have absolute lymphopenia. Absolute lymphopenia is an important marker for SCID, but this finding is not present in all SCID newborns such as the presence of maternal lymphocytes or B-cells.^[33, 34]^ Also, the number of lymphocytes in a newborn can be affected by many conditions.^[33, 35]^ Therefore, we did not report newborns with transient lymphopenia in our findings.

We were able to use NGS for genetic analysis in one newborn only. We found a missense mutation in NFKB2 gene. NFKB2 is responsible for the activation of genes related to inflammation, immune function, bone metabolism and oncogenesis.^[36]^ It is reported in the literature that patients with deficient NFKB2 function show common variable immunodeficiency (CVID) like features. Such patients were characterized by early-onset CVID associated with autoimmunity, reduction in circulating B-cells, adrenocorticotropic hormone deficiency, hair and/or nail disorders and occasional other pituitary hormone deficiencies.^[36, 37]^ However, we found no abnormal immunological laboratory findings, although we followed the newborn with NFKB2 mutation in our cohort until the age of 3. We attributed frequent respiratory tract infection to his neurological condition. In the literature, it has been reported that a patient with NFKB2 nonsense mutation may not have any clinical findings.^[37]^ To our knowledge, this is the first newborn with NFKB2 mutation reported in the literature by NBS.

PIDs are common in our country than western societies. The reason for the high prevalence could be explained by founder mutation effects due to high consanguinity rates at least in some patients.^[12]^ It is clear that early detection of PIDs will prevent unnecessary investigations and prolonged complications and will contribute to the country's economy by avoiding unnecessary health expenditures especially in our country. There are some limitations of the present study. Firstly, we could not perform immunological investigations on most of the newborns with positive screening results due to insufficient contact information in the medical file. The incidence of T-cell lymphopenia that we have found may actually be high. Secondly, we did not calculate the cutoff values of TRECs and KRECs for our country. Third limitation is that our newborn cohort was a small. Similar studies should be conducted in a higher number of newborn cohorts and under better follow-up in our country.

## Conclusion

In this study, NBS for severe PIDs through TRECs and KRECs assays was performed for the first time in our country. Although we could not find a SCID newborn, we found more non-SCID T-cell lymphopenia than we expected. We advise that combined TRECs and KRECs assay should be considered for routine NBS program in our country. Studies involving more newborns should be conducted to detect SCID in our country.

### Disclosures

**Ethics Committee Approval:** This study was approved by local Ethical Committee (No: 2014/678) and the Turkish Ministry of Health, Public Hospitals Authority, Turkey (No: 54103609/604.02).

**Peer-review:** Externally peer-reviewed.

**Conflict of Interest:** None declared.

**Authorship Contributions:** Concept – A.Y., G.O.; Design – S.K., C.A., G.O., A.Y.; Supervision – S.K., M.K.A., C.A., G.O., A.Y.; Materials – S.K., M.K.A., E.O.O., D.Y., H.B.; Data collection &/or processing – S.K., M.K.A., G.H.; Analysis and/or interpretation – S.K., M.K.A., G.H., H.B., A.Y.; Literature search – S.K.; Writing – S.K., M.K.A.; Critical review – S.K., M.K.A.

## References

[1] Picard C, Bobby Gaspar H, Al-Herz W, Bousfiha A, Casanova JL, Chatila T (2018). International Union of Immunological Societies: 2017 primary immunodeficiency diseases committee report on inborn errors of ımmunity.. J Clin Immunol.

[2] King JR, Hammarström L (2018). Newborn screening for primary immunodeficiency diseases: history, current and future practice.. J Clin Immunol.

[3] Puck JM (2019). Newborn screening for severe combined immunodeficiency and T-cell lymphopenia.. Immunol Rev.

[4] van der Burg M, Mahlaoui N, Gaspar HB, Pai SY (2019). Universal newborn screening for severe combined immunodeficiency (SCID).. Front Pediatr.

[5] Dorsey MJ, Dvorak CC, Cowan MJ, Puck JM (2017). Treatment of infants identified as having severe combined immunodeficiency by means of newborn screening.. J Allergy Clin Immunol.

[6] Wilson JM, Jungner YG (1968). Principles and practice of mass screening for disease.. Bol Oficina Sanit Panam.

[7] Borte S, von Döbeln U, Fasth A, Wang N, Janzi M, Winiarski J (2012). Neonatal screening for severe primary immunodeficiency diseases using high-throughput triplex real-time PCR.. Blood.

[8] Borte S, von Döbeln U, Hammarström L (2013). Guidelines for newborn screening of primary immunodeficiency diseases.. Curr Opin Hematol.

[9] Chan K, Puck JM (2005). Development of population-based newborn screening for severe combined immunodeficiency.. J Allergy Clin Immunol.

[10] van Zelm MC, Szczepanski T, van der Burg M, van Dongen JJ (2007). Replication history of B lymphocytes reveals homeostatic proliferation and extensive antigen-induced B cell expansion.. J Exp Med.

[11] Nakagawa N, Imai K, Kanegane H, Sato H, Yamada M, Kondoh K (2011). Quantification of κ-deleting recombination excision circles in Guthrie cards for the identification of early B-cell maturation defects.. J Allergy Clin Immunol.

[12] El-Sayed ZA, Radwan N (2020). Newborn screening for primary immunodeficiencies: the gaps, challenges, and outlook for developing countries.. Front Immunol.

[13] Kwan A, Abraham RS, Currier R, Brower A, Andruszewski K, Abbott JK (2014). Newborn screening for severe combined immunodeficiency in 11 screening programs in the United States.. JAMA.

[14] de Felipe B, Olbrich P, Lucenas JM, Delgado-Pecellin C, Pavon-Delgado A, Marquez J (2016). Prospective neonatal screening for severe T- and B-lymphocyte deficiencies in Seville.. Pediatr Allergy Immunol.

[15] Kilic SS, Ozel M, Hafizoglu D, Karaca NE, Aksu G, Kutukculer N (2013). The prevalences [correction] and patient characteristics of primary immunodeficiency diseases in Turkey--two centers study.. J Clin Immunol.

[16] Thomas C, Durand-Zaleski I, Frenkiel J, Mirallié S, Léger A, Cheillan D (2019). Clinical and economic aspects of newborn screening for severe combined immunodeficiency: DEPISTREC study results.. Clin Immunol.

[17] van der Spek J, Groenwold RH, van der Burg M, van Montfrans JM (2015). TREC based newborn screening for severe combined immunodeficiency disease: a systematic review.. J Clin Immunol.

[18] Shearer WT, Dunn E, Notarangelo LD, Dvorak CC, Puck JM, Logan BR (2014). Establishing diagnostic criteria for severe combined immunodeficiency disease (SCID), leaky SCID, and Omenn syndrome: the Primary Immune Deficiency Treatment Consortium experience.. J Allergy Clin Immunol.

[19] Modell V, Orange JS, Quinn J, Modell F (2018). Global report on primary immunodeficiencies: 2018 update from the Jeffrey Modell Centers Network on disease classification, regional trends, treatment modalities, and physician reported outcomes.. Immunol Res.

[20] Amatuni GS, Currier RJ, Church JA, Bishop T, Grimbacher E, Nguyen AA (2019). Newborn screening for severe combined immunodeficiency and T-cell lymphopenia in California, 2010-2017.. Pediatrics.

[21] Argudo-Ramírez A, Martín-Nalda A, Marín-Soria JL, López-Galera RM, Pajares-García S, González de Aledo-Castillo JM (2019). First universal newborn screening program for severe combined immunodeficiency in Europe.. Two-years' experience in Catalonia (Spain). Front Immunol.

[22] Routes JM, Grossman WJ, Verbsky J, Laessig RH, Hoffman GL, Brokopp CD (2009). Statewide newborn screening for severe T-cell lymphopenia.. JAMA.

[23] Barbaro M, Ohlsson A, Borte S, Jonsson S, Zetterström RH, King J (2017). Newborn screening for severe primary ımmunodeficiency diseases in Sweden-a 2-year pilot TREC and KREC screening study.. J Clin Immunol.

[24] Modell V, Knaus M, Modell F (2014). An analysis and decision tool to measure cost benefit of newborn screening for severe combined immunodeficiency (SCID) and related T-cell lymphopenia.. Immunol Res.

[25] Nourizadeh M, Shakerian L, Borte S, Fazlollahi M, Badalzadeh M, Houshmand M (2018). Newborn screening using TREC/KREC assay for severe T and B cell lymphopenia in Iran.. Scand J Immunol.

[26] Remaschi G, Ricci S, Cortimiglia M, De Vitis E, Iannuzzi L, Boni L TREC and KREC in very preterm infants: reference values and effects of maternal and neonatal factors.. J Matern Fetal Neonatal Med.

[27] Kanegae MPP, Barreiros LA, Sousa JL, Brito MAS, Oliveira EB, Soares LP (2017). Newborn screening for severe combined immunodeficiencies using trecs and krecs: second pilot study in Brazil.. Rev Paul Pediatr.

[28] Olbrich P, de Felipe B, Delgado-Pecellin C, Rodero R, Rojas P, Aguayo J (2014). A first pilot study on the neonatal screening of primary immunodeficiencies in Spain: TRECS and KRECS identify severe T- and B-cell lymphopenia.. [Article in Spanish]. An Pediatr (Barc).

[29] Rechavi E, Lev A, Lee YN, Simon AJ, Yinon Y, Lipitz S (2015). Timely and spatially regulated maturation of B and T cell repertoire during human fetal development.. Sci Transl Med.

[30] Odabasi IO, Bulbul A (2020). Neonatal sepsis.. Sisli Etfal Hastan Tip Bul.

[31] Rechavi E, Lev A, Simon AJ, Stauber T, Daas S, Saraf-Levy T (2017). First year of Israeli newborn screening for severe combined immunodeficiency-clinical achievements and insights.. Front Immunol.

[32] Shakerian L, Pourpak Z, Shamlou S, Domsgen E, Kazemnejad A, Dalili H (2019). Determining laboratory reference values of TREC and KREC in different age groups of Iranian healthy individuals.. Iran J Allergy Asthma Immunol.

[33] El-Sayed SS, El-Sayed Afifi HM, Yahia SA (2013). Neonatal screening for absolute lymphopenia.. Egypt J Pediatr Allergy Immunol.

[34] Buckley RH (2002). Primary immunodeficiency diseases: dissectors of the immune system.. Immunol Rev.

[35] Puck JM (2012). Laboratory technology for population-based screening for severe combined immunodeficiency in neonates: the winner is T-cell receptor excision circles.. J Allergy Clin Immunol.

[36] Kotlinowski J, Bukowska-Strakova K, Koppolu A, Kosińska J, Pydyn N, Stawinski P (2019). A novel monoallelic nonsense mutation in the NFKB2 gene does not cause a clinical manifestation.. Front Genet.

[37] Klemann C, Camacho-Ordonez N, Yang L, Eskandarian Z, Rojas-Restrepo JL, Frede N (2019). Clinical and immunological phenotype of patients with primary immunodeficiency due to damaging mutations in NFKB2.. Front Immunol.

